# Myriad, Microscopic and Marvelous: The World of Antoni van Leeuwenhoek

**DOI:** 10.3201/eid3206.260353

**Published:** 2026-06

**Authors:** Jonathan H. Sogin

**Keywords:** Antoni van Leeuwenhoek, microscopes, books and media

In Myriad, Microscopic and Marvellous: The World of Antoni van Leeuwenhoek, Dutch historian and journalist Geertje Dekkers invites readers to examine the life of the “father of microbiology,” Antoni (sometimes spelled Antony or Antonie) van Leeuwenhoek (1632–1723), and the rudimentary practices by which he observed, theorized, and reported on plants, “little animals” (now known to be microorganisms), spermatozoa, and other specimens in the 17th and 18th Centuries (Figure). Although Dekkers contextualizes some of van Leeuwenhoek’s work with contemporary knowledge, the book primarily describes his atypical entry into scientific research, his motivations for conducting research, and his interactions with the broader scientific community. The author grounds readers in thorough historical background, citing several research letters written by van Leeuwenhoek (among other sources), but provides space for readers to speculate on what van Leeuwenhoek observed, how he felt, and how his environment shaped his work. In doing so, Dekkers situates readers in a largely unfamiliar world, one in which the concepts and vocabulary to describe unicellular organisms had not yet emerged.

**Figure:**
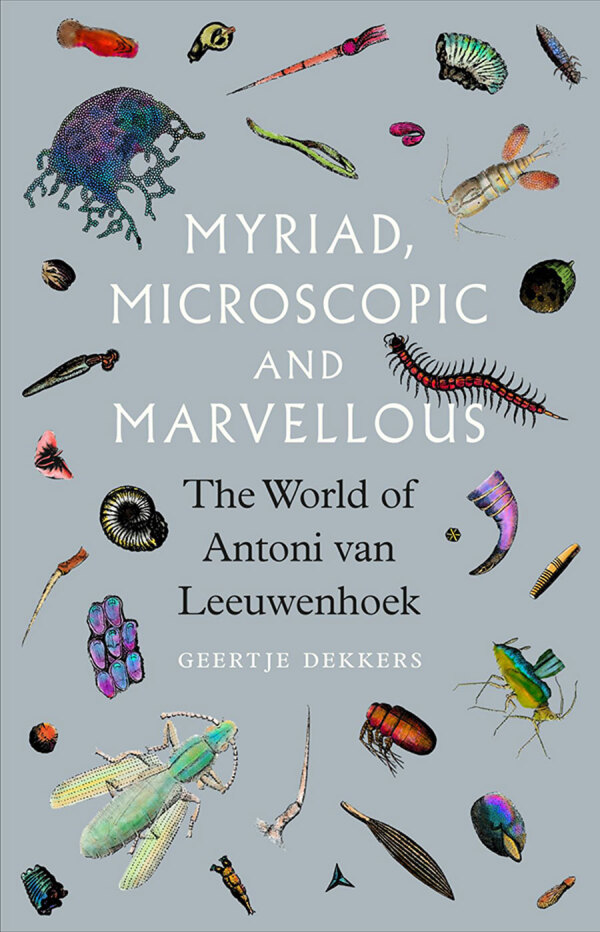
Myriad, Microscopic and Marvelous: The World of Antoni van Leeuwenhoek.

Van Leeuwenhoek spent most of his life in Delft, Dutch Republic, where he worked first as a textile merchant and later as a municipal civil servant. Despite lacking academic training, he began conducting microscopy research, driven by personal curiosity and the work of other scientists. Although unrecognizable by modern standards, the microscopes van Leeuwenhoek made were superior to those made by others, capable of greater magnification and clarity because of their single-lens construction. That advantage enabled him to make more accurate and detailed observations of his specimens, which were the underpinning of his scientific legacy. In 1673, van Leeuwenhoek’s early work was sent to the Royal Society of England and published in the society’s official journal, Philosophical Transactions. He continued publishing research in the journal until his death in 1723, including the first descriptions of microorganisms, although he did not know what they were. Dekkers emphasizes that van Leeuwenhoek “had no notion of the composition of the creatures, how they lived, and the impact they had on their environments… They were interesting to look at, but that was all.” He was elected a fellow to the Society in 1680.

The book elicits the comparison of the scientific process from van Leeuwenhoek’s era to today. Van Leeuwenhoek, an untrained person driven by often misguided theories, used homemade contraptions to look at (at the time) incomprehensibly small creatures with limited reproducibility. Yet his research was widely recognized, and his observations revolutionized the field of biology. Could someone like van Leeuwenhoek succeed today? Historical successes such as van Leeuwenhoek’s raise questions about whether a self-driven, self-taught citizen scientist could achieve such revolutionary success in today’s research landscape.

Ultimately, readers can expect to gain a better understanding of both van Leeuwenhoek’s legacy and the nature of scientific inquiry itself. Dekkers draws readers into van Leeuwenhoek’s world in an engaging and accessible biographical narrative, featuring 58 illustrations drawn by van Leeuwenhoek and others. Readers need no technical knowledge to understand its content, but those with research experience may have greater appreciation for the unstructured environment in which he worked.

